# Large-Pore Platelet-Rich Fibrin with a Mg Ring to Allow MC3T3-E1 Preosteoblast Migration and to Improve Osteogenic Ability for Bone Defect Repair

**DOI:** 10.3390/ijms22084022

**Published:** 2021-04-14

**Authors:** Pei-Chun Wong, Chen-Yun Wang, Jason Shian-Ching Jang, Chian-Her Lee, Jia-Lin Wu

**Affiliations:** 1 Department of Orthopedics, School of Medicine, College of Medicine, Taipei Medical University, Taipei 110, Taiwan; pcwong0424@tmu.edu.tw (P.-C.W.); chianherlee@yahoo.com.tw (C.-H.L.); 2 Department of Orthopedics, Taipei Medical University Hospital, Taipei 110, Taiwan; 3 Orthopedics Research Center, Taipei Medical University Hospital, Taipei 110, Taiwan; 4 School of Biomedical Engineering, College of Biomedical Engineering, Taipei Medical University, Taipei 110, Taiwan; amber16743@tmu.edu.tw; 5 Institute of Materials Science and Engineering, National Central University, Taoyuan 33043, Taiwan; jscjang@ncu.edu.tw; 6 Department of Mechanical Engineering, National Central University, Taoyuan 33043, Taiwan

**Keywords:** repeated freeze drying method, large-pore platelet-rich fibrin, Mg ring, degradation, migration, calcium deposition, bone defect

## Abstract

Platelet-rich fibrin (PRF) is a natural fibrin meshwork material with multiple functions that are suitable for tissue engineering applications. PRF provides a suitable scaffold for critical-size bone defect treatment due to its platelet cytokines and rich growth factors. However, the structure of PRF not only promotes cell attachment but also, due to its density, provides a pool for cell migration into the PRF to facilitate regeneration. In our study, we used repeated freeze drying to enlarge the pores of PRF to engineer large-pore PRF (LPPRF), a type of PRF that has expanded pores for cell migration. Moreover, a biodegradable Mg ring was used to provide stability to bone defects and the release of Mg ions during degradation may enhance osteoconduction and osteoinduction. Our results revealed that cell migration was more extensive when LPPRF was used rather than when PRF was used and that LPPRF retained the growth factors present in PRF. Moreover, the Mg ions released from the Mg ring during degradation significantly enhanced the calcium deposition of MC3T3-E1 preosteoblasts. In the present study, a bone substitute comprising LPPRF combined with a Mg ring was demonstrated to have much potential for critical-size bone defect repair.

## 1. Introduction

Bone defect repair is a common regenerative procedure in orthopedics. Bone defects may be caused by high-energy trauma or tumors that remove a large portion of bone tissue [1,2] and they occur mostly at the cranium, ulna, radius, tibia and femur [3,4,5,6,7]. Traditionally, clinics have three treatment options for critical-size bone defects: (1) autologous bone graft transplantation, (2) allogeneic bone graft transplantation and (3) synthetic bone substitute transplantation [8,9,10]. However, these methods have some disadvantages, such as donor site complications, donor site instability and slow healing at the transplantation site. Several approaches have been investigated for critical-size bone defects, including bioactive scaffold introduction, stem cells, growth factors and gene therapy for enhancing bone regeneration and bone healing [1,11]. However, autologous bone graft transplantation remains the optimal treatment for long bone defects. Other therapeutic strategies lack the ideal composite graft, appropriate drug delivery system, appropriate materials, suitable cells and compatible complex system growth factors [1].

PRF is a natural fibrin meshwork material obtained from human blood through the centrifugation process under a specific centrifuge duration and specific gravity. PRF mostly comprises platelet cytokines and rich growth factors [12,13], which are essential for tissue repair and regeneration. Platelets are mediated to form blood clots in the wound healing process and to release growth factors [14]. Many studies have reported that PRF contains effective growth factors—such as vascular endothelial growth factor (VEGF), insulin-like growth factor-1 (IGF-1), transforming growth factor beta (TGF-β) and platelet-derived growth factor—for tissue repair and regeneration [15,16,17,18], various studies in vivo are exploring the use of PRF in tissue and bone regeneration [19]. Additionally, these growth factors neither decay nor denature after undergoing freeze drying [16]. However, the dense structure of PRF limits the capacity of cells to migrate to, in turn, complete PRF. Moreover, a mineralized matrix can be generated only on the surface of PRF, which is the leading cause of the failure to repair long bone defects. Additionally, the low mechanical strength of PRF as a scaffold for long bone repair causes structural instability in load-bearing bone tissue when loading occurs in everyday activity. This problem is not inconsequential because muscular and load stresses, as well as mobility, can affect postoperative outcomes [20].

Ti-based materials are the most commonly used bioinert materials for orthopedic and dental implants. They can provide mechanical properties that are suited to human bones for bone fixation. However, the use of Ti implants requires secondary surgery where Ti implants are removed after the bones completely heal. Mg-based materials have been extensively investigated due to their characteristics, such as their biocompatibility, biodegradability, osteogenic abilities and similar Young’s modulus to that of bone, which renders them suitable for bone implant applications. In particular, Mg ions released from Mg-based material during degradation are key to enhancing osteoconductive, osteoinductive and osteogenic properties.

**Figure 1:**
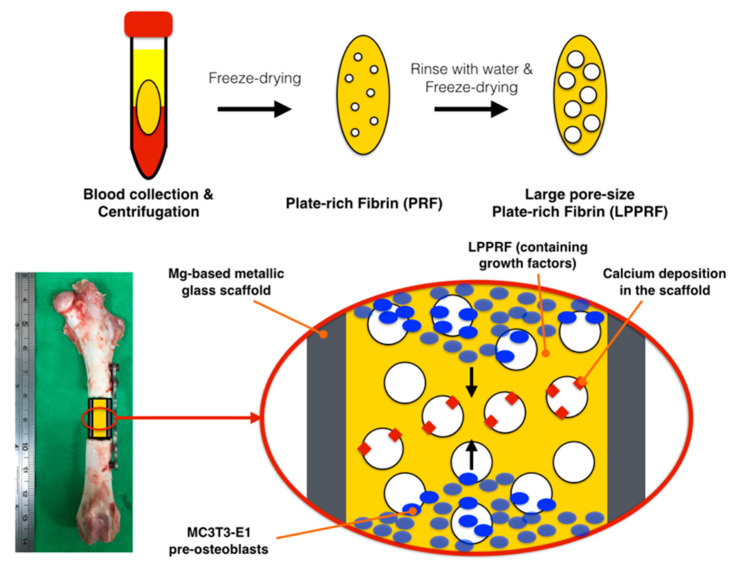
Large-pore platelet-rich fibrin and a magnesium scaffold improved cell migration and osteogenic differentiation for critical-size bone defect repair.

In the present study, the pore sizes of PRF were first enlarged through a repeated application of the freeze drying method to create an engineered PRF known as large-pore PRF (LPPRF). The Mg ring, which can be weight-bearing was then combined with LPPRF to become a bioactive scaffold for bone defects. We focused on the investigation of the cell migration in LPPRF and the interaction with the Mg ring to observe signs of calcium deposition (Figure 1).

## 2. Results

### 2.1. Fundamental Properties of Modified PRF

**Figure 2:**
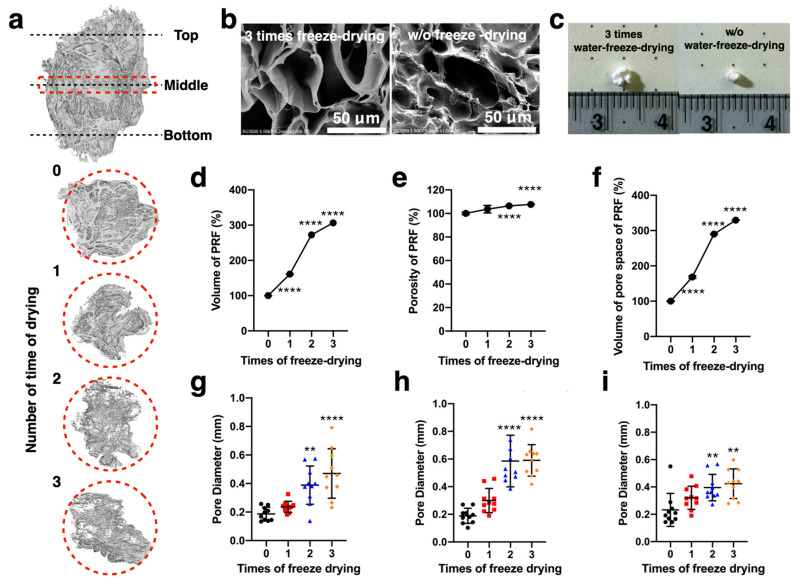
Platelet-rich fibrin scaffold preparation and analysis. After collection, the platelet-rich fibrin was subjected to repeated freeze drying to remove the water naturally present within it. It was then immersed in water and dried using three cycles of the repeated freeze drying method to expand the pores, thus enabling cell migration and ingrowth. Large-pore platelet-rich fibrin was thereby produced. (**a**) Micro-CT images of platelet-rich fibrin after several iterations of freeze drying; (**b**) scanning electron microscopy images of platelet-rich fibrin; (**c**) images of platelet-rich fibrin; (**d**) volume of platelet-rich fibrin; (**e**) porosity of platelet-rich fibrin; (**f**) volume of platelet-rich fibrin pores; (**g**) pore diameter of top-section platelet-rich fibrin; (**h**) pore diameter of middle-section platelet-rich fibrin; and (**i**) pore diameter of bottom-section platelet-rich fibrin. (Individual data points are shown as means ± SD; N = 5 per group; ** *p* < 0.01 and **** *p* < 0.001).

In Figure 2a, the LPPRF, which was created through the repeated freeze drying process, had a volume visibly greater than that of the PRF displayed. We first used a micro-CT scan to analyze the volume and porosity of the PRF and LPPRF and determine the volume of their pores. The PRF and LPPRF pores are detailed in Figure 2b. In the macro view, the LPPRF was observed to be swollen due to the repeated freeze drying (Figure 2c). The quantitative results revealed that the volume, porosity and pore volume of the LPPRF were significantly larger than those of the PRF. These reliable results indicated that repeated freeze drying easily and effectively modified the structure and increased the pore size (Figure 2d–f). Moreover, the diameter of the pores indicated a significant difference between the PRFs before and after undergoing the freeze drying process (Figure 2g–i).

**Figure 3:**
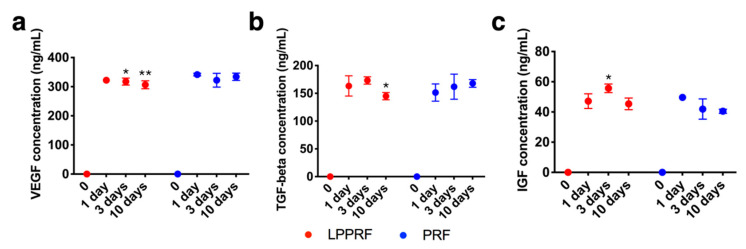
Growth factors released from platelet-rich fibrin before and after repeated freeze drying: (**a**) vascular endothelial growth factor (VEGF), (**b**) insulin-like growth factor-1 (IGF-1) and (**c**) transforming growth factor beta (TGF-β). (Individual data points are shown as means ± SD; N = 5 per group; * *p* < 0.05 and ** *p* < 0.01).

Growth factors released to phosphate-buffered saline (PBS) from PRF and LPPRF were detected and quantified by using an enzyme-linked immunosorbent assay (ELISA) kit. VEGF, TGF-β and IGF were detected in the PBS for both PRF and LPPRF. Growth factors were detected in the PBS even after 10 days and the release profiles of PRF and LPPRF were similar (Figure 3). The growth factor content of LPPRF was thus, unaffected by repeated freeze drying.

### 2.2. Degradation Behavior of Metallic Rings

**Figure 4:**
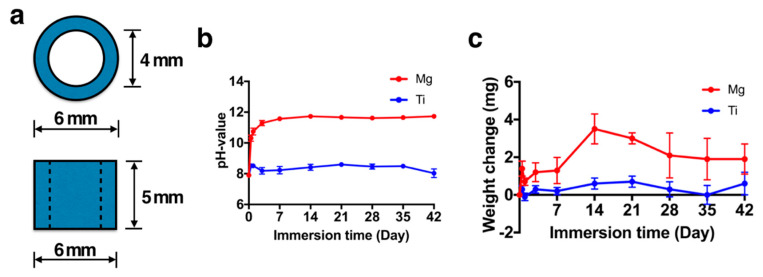
Degradation behavior of the pure magnesium ring: (**a**) dimensions of the metal ring, (**b**) weight change of the pure magnesium ring, and (**c**) pH change of Hank’s solution. (N = 5 per group).

**Figure 5:**
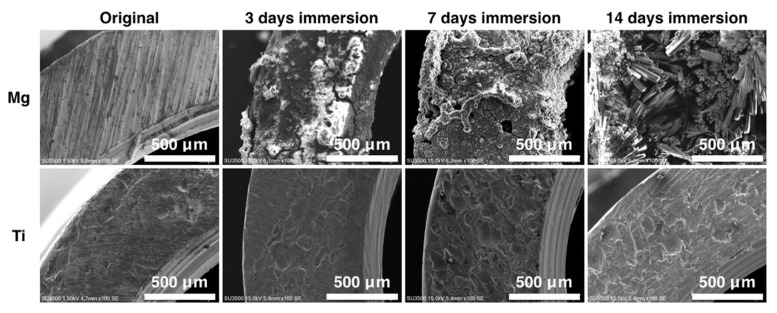
Surface morphology of magnesium and titanium rings after 3, 7 and 14 days of immersion in Hank’s solution.

The dimensions of the Mg and Ti rings are presented in Figure 4a. The volume of Hank’s solution used was selected in accordance with the surface area of the metallic rings. The pH of the Hank’s solution in which the Mg rings were immersed was increased to 9 after 4 h of degradation and then increased to 11 for the subsequent 3 days’ immersion. However, the Hank’s solution in which the Ti rings were immersed was maintained at a neutral pH (Figure 4b). The weight of the Ti rings changed little during 42 days of immersion (Figure 4c). However, the weight of the Mg rings increased until day 14 of immersion due to the formation of degradation products (Figure 5). After day 14, the weight of the Mg rings began to decrease due to the increased degradation rate, which was much higher than the rate of degradation product generation (Figure 4c). The surface topology of the Mg ring and Ti ring could be clearly observed (Figure 5). The quantity of degradation product on the Mg ring increased with immersion time. The surface of the Ti ring exhibited no change until 14 days after immersion.

### 2.3. Cell Viability of MC3T3-E1 Preosteoblasts Treated with PRF, LPPRF, Mg Ring and Ti Ring Precipitate Media and Corresponding Mixtures

**Figure 6:**
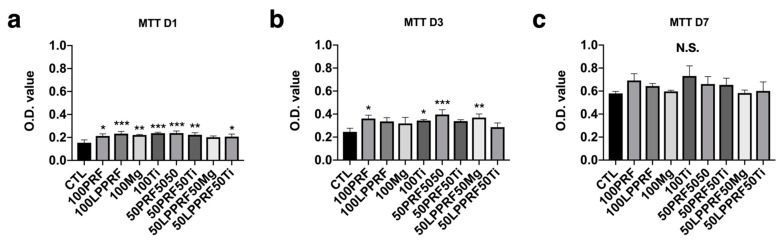
Cell proliferation of MC3T3-E1 preosteoblasts cultured with platelet-rich fibrin, large-pore platelet-rich fibrin, magnesium ring and titanium ring precipitate media and their mixtures for (**a**) 1, (**b**) 3 and (**c**) 7 days. (N = 5 per group; * *p* < 0.05, ** *p* < 0.01, and *** *p* < 0.005).

The cell viabilities of MC3T3-E1 preosteoblasts cultured with 100% PRF medium, 100% LPPRF medium, 100% Mg medium, 100% Ti medium, 50% PRF + 50% Mg medium, 50% PRF + 50% Ti medium, 50% LPPRF + 50% Mg medium and 50% LPPRF + 50% Ti medium are presented in Figure 6. After 1 day of incubation, the cell viabilities of all groups were much higher than that of the control group. However, until 7 days of incubation, the cell viabilities of the precipitate medium groups were still higher but much closer to that of the control group.

### 2.4. Migration Capacity of MC3T3-E1 Preosteoblasts Treated with PRF, LPPRF, Mg Ring and Ti Ring Precipitate Media and Their Mixtures

**Figure 7:**
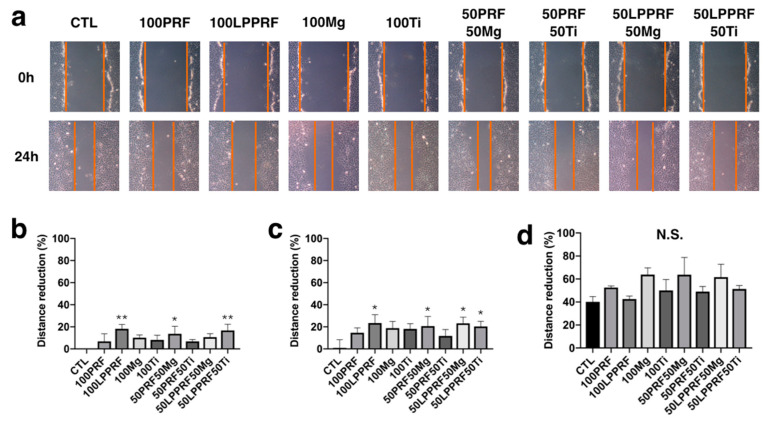
Migration capacity of MC3T3-E1 preosteoblasts cultured with platelet-rich fibrin, large-pore platelet-rich fibrin, magnesium ring and titanium ring precipitate media and their mixtures. (**a**) Cell migration at first scratch and after 24 h of incubation; reduction of the gap due to migration of MC3T3-E1 preosteoblasts after (**b**) 4, (**c**) 8 and (**d**) 24 h of incubation. (N = 5 per group; * *p* < 0.05 and ** *p* < 0.01).

To treat a long-distance bone defect, we first used the precipitate media from Mg and Ti rings, PRF and LPPRF to test the migration capacity of MC3T3-E1 preosteoblasts. Figure 7a shows the migration of MC3T3-E1 osteoblasts after scratching and 24 h after incubation. The gap due to the migration of MC3T3-E1 preosteoblasts was measured with Image J software and normalized to that for the control. After 4 h of incubation, the 100% LPPRF medium had a most greatly reduced gap (Figure 7b). Compared with the control, 100% LPPRF medium, 50% PRF + 50% Mg medium, 50% LPPRF + 50% Mg medium and 50% LPPRF + 50% Ti medium had a greatly reduced gap after 8 h of incubation (Figure 7c). However, no significant difference between the medium groups was measured until after 24 h of incubation (Figure 7d). PRF and LPPRF seemingly enhanced cell migration capacity in the early stages of cell migration.

### 2.5. Migration Capacity of MC3T3-E1 Preosteoblasts on PRF and LPPRF Sheets

**Figure 8:**
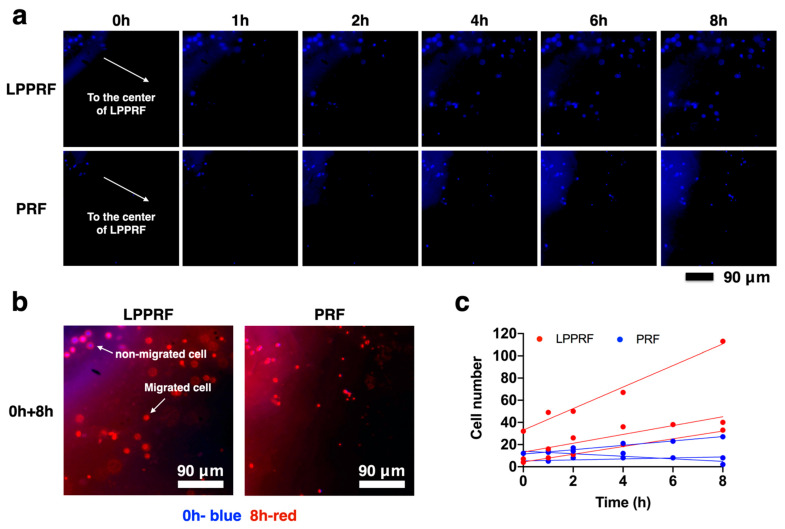
Migration capacity of MC3T3-E1 preosteoblasts cultured in large-pore platelet-rich fibrin membrane and platelet-rich fibrin membrane for 8 h. (**a**) Images of migrated cells stained with Hoechst 33,342 in large-pore platelet-rich fibrin and platelet-rich fibrin at 0, 1, 2, 4, 6 and 8 h after seeding, (**b**) Combination of the images obtained at 0 and 8 h showing cell migration in large-pore platelet-rich fibrin and platelet-rich fibrin and (**c**) Number of migrated cells against time.

According to our hypothesis, LPPRF can achieve greater cell migration than can PRF, which has smaller pores than LPPRF. Figure 8a indicates that the MC3T3-E1 preosteoblasts that were cultured, attached and migrated in the fibrin structure of LPPRF and PRF sheets at 1, 2, 4, 6 and 8 h. Images were captured continually at the same position using a fluorescence microscope. After 24 h of incubation, the cells had migrated in the scaffold and were visible in the images. Both the LPPRF and PRF sheets exhibited cell migration from the sheet edge to the center. To clearly identify the differences in cell locations between 0 and 8 h of incubation, the color of the 8 h images was changed to red by using Image J software and the images captured at the two time points were overlaid (Figure 8b). In Figure 8b, the blue labels represent the cells that first attached in the LPPRF and PRF sheets and the red labels represent the cells that migrated after 8 h. The cells attached from the beginning were not observed to have migrated to other positions. However, many cells migrated to another position within the view that we captured. At each time point, the cell number was counted to establish cell migration ability in LPPRF and PRF. Figure 8c presents a plot of cell number against time and the assistance trend line clearly indicates the extent of cell migration. LPPRF yielded superior cell migration ability for MC3T3-E1 preosteoblasts relative to PRF.

### 2.6. Cell Morphology of MC3T3-E1 Preosteoblasts on PRF and LPPRF

**Figure 9:**
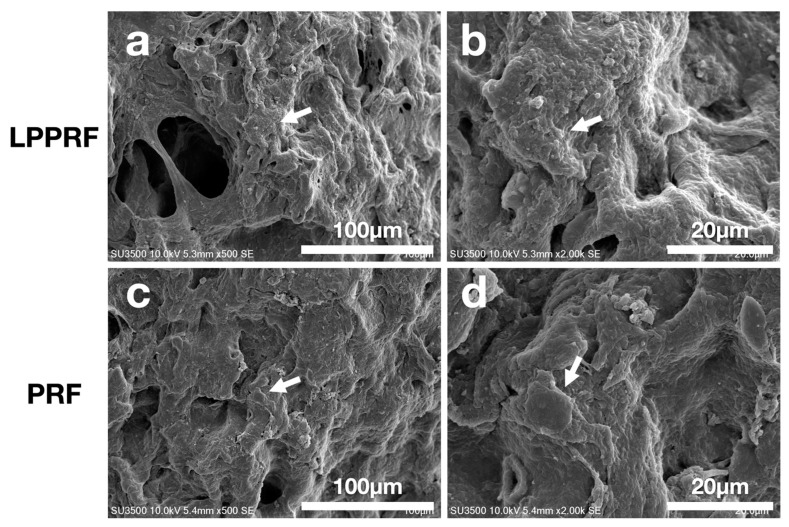
Scanning electron microscopy images of MC3T3-E1 preosteoblasts cultured on (**a**,**b**) large-pore platelet-rich fibrin and (**c**,**d**) platelet-rich fibrin captured with different magnification.

The cell adhesion morphologies in LPPRF and PRF were captured using scanning electron microscopy after fixation and dehydration (Figure 9). Both LPPRF and PRF provided suitable surfaces for cell adhesion. The cytoskeleton of MC3T3-E1 preosteoblasts was expanded on LPPRF and the formation of pseudopodia was noted. Pronounced spreading with a distinct spindle shape occurred and it was well attached to the surface of LPPRF. By contrast, the aspect of MC3T3-E1 preosteoblasts exhibited a round shape, which reflected poor cell attachment. Compared with PRF, LPPRF provided a surface that was more suitable for cell adhesion.

### 2.7. Calcium Deposition of MC3T3-E1 Preosteoblasts Treated with PRF, LPPRF, Mg Ring and Ti Ring Precipitate Media and Their Mixtures

**Figure 10:**
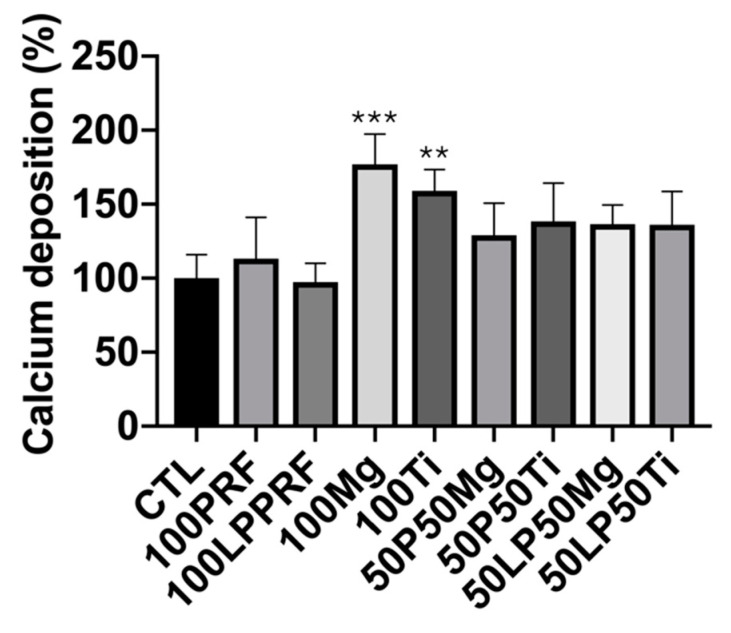
Calcium deposition with platelet-rich fibrin, large-pore platelet-rich fibrin, magnesium ring and titanium ring precipitate media and their mixtures. (N = 5 per group; ** *p* < 0.01 and *** *p* < 0.005).

The extracellular matrix calcification of MC3T3-E1 preosteoblasts precultured with PRF, LPPRF, Mg ring and Ti ring precipitate media and their mixtures was detected through alizarin red S staining. The quantitative results of abstract alizarin red S staining are presented in Figure 10. Compared with that in the control group, calcium deposition was enhanced in most of the experimental groups. Moreover, Mg and Ti precipitate media substantially enhanced the calcium deposition, specifically a 170% increase compared with the control.

### 2.8. Calcium Deposition of MC3T3-E1 Preosteoblasts on PRF and LPPRF

**Figure 11:**
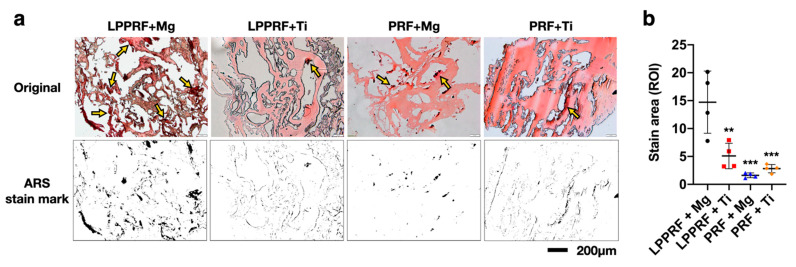
(**a**) Calcium deposition of MC3T3-E1 preosteoblasts on platelet-rich fibrin and large-pore platelet-rich fibrin with magnesium and titanium rings, as detected through alizarin red S staining on the scaffold with 5-μm-thick sections. The location of calcium deposits is indicated by a yellow arrow. (**b**) Quantitative results of stain marks, obtained using Image J software. (N = 4 per group; ** *p* < 0.01, and *** *p* < 0.005).

To detect the extracellular matrix calcification of MC3T3-E1 preosteoblasts deposited on PRF and LPPRF with Mg and Ti rings, we used the histological method of alizarin red S staining. The stain marks in Figure 11a represent calcium deposition, as indicated by the yellow arrow. Under the same magnification, the LPPRF + Mg medium group demonstrated much more stain marks than the other groups did. Additionally, the stain marks obtained for the LPPRF + Mg group were dispersed across the LPPRF, whereas the stain marks for the LPPRF + Ti, PRF + Ti and PRF + Mg media were located in a partial region of the PRF and LPPRF. Figure 11b presents the quantitative results of calcium deposition marks. The LPPRF + Mg precipitate media exhibited the highest calcium deposition area compared with the other groups; additionally, no significant difference between each group except LPPRF + Mg, was observed.

## 3. Discussion

PRF has been widely used in tissue engineering due to its excellent biocompatibility, growth factor content, ease of collection and ability to be produced by the human body. The network structure of PRF not only offers a surface that is suitable for cell attachment but also ensures a high potential for cell migration through the release of growth factors [21]. PRF has great potential for use with the scaffolds used in tissue engineering. However, the dense network structure of PRF renders cell migration difficult, thus limiting its application for long-range tissue defects or large-volume tissue defects, such as critical-size bone defects. In the present study, the freeze drying method provided a safe and straightforward means of enlarging the pores of the network structure. The fundamental laws of physics dictate that the volume of water is greater when frozen than when liquified. Moreover, when the freeze drying method is employed, the growth factors in the material are retained [22].

The size of pores in PRF can be adjusted by changing the number of times it is freeze dried and the pore size of PRF can thus be engineered and controlled. The overall pore size nonetheless depends on the initial PRF pore size and the sizes of single pores cannot be adjusted. Compared with synthetic material, natural PRF exhibits a superior biological response, providing advantages in clinical applications. PRF pore size can be adjusted and modified for various applications and sites. In orthopedic applications, the PRF pore size can be modified in accordance with the size of bone cells. Cell size, cell attachment, scaffold pore size and surface topography are four key factors that affect cell migration across membranes [23]. However, lot-to-lot variability, lack of sourcing, potential contaminations and the high cost of PRF are its inherent disadvantages. Li et al. analyzed the migration of human umbilical cord-derived mesenchymal stem cells across a polycarbonate membrane with pore sizes of 0.4, 3 and 8 μm; their analysis determined the migration to be 0%, 1.8% and 8%, respectively [24]. Peyton et al. analyzed the migration of mesenchymal stem cells across a membrane scaffold comprising poly(ethylene glycol) and with pore sizes of 7, 12 and 17 μm; the optimal results were obtained from the intermediate-sized (12 μm) pores [25]. According to Bružauskaitė et al., scaffolds with pore sizes ranging from 50 nm to 12 μm regulate cellular attachment, cell–cell interaction and migration across the membrane [23]. However, scaffolds with large pores (diameters of approximately 100 μm or greater) have stronger tissue regeneration functionalities. Murphy et al. investigated the relationship between collagen–glucosaminoglycan scaffolds with pore sizes of 85, 120 and 325 μm and the adhesion and differentiation of osteoblasts. After 48 h of incubation, the scaffold with 120 μm pores exhibited the most favorable cell adhesion and proliferation. On day 7, the cell count of osteoblasts was highest on the scaffold with 325 μm pores. The scaffold with 100 μm pores was suitable for cell adhesion and proliferation, whereas cell migration occurred faster on the scaffold with 300 μm pores [26]. In the tissue engineering strategy, the regeneration process must be activated after cell migration and attachment. In numerous studies of bone regeneration, the pore size of the scaffold has ranged from 100 μm to more than 300 μm. A pore size of approximately 100 μm benefits the induction of osteochondral formation under hypoxic conditions before osteogenesis; moreover, large pores (>300 μm) may directly induce osteogenesis [27].

Mg and its alloys have been widely investigated due to their biocompatibility, their degradability and the similarity of their mechanical properties to those of bone and for the fact that their release of Mg ions can enhance osteoconduction and osteoinduction [28,29]. In other studies, Mg-based materials have been used as fixation implants or bone substitutes. In the present study, the Mg ring not only offered mechanical support for bone defects but also enhanced calcium deposition. Additionally, the surface roughness of the Mg ring changed during the process of degradation and the generation of corrosion products. Cell migration and cell deposition were considerably different between each stage of surface roughness [30]. The rougher surface can enhance the cell migration capacity. In the pH-value measurement, the pH-value of Hank’s solution increased rapidly from the initial measurement. This pH change profile has been reported in many studies and is similar to our results; however, in vivo studies have not shown that the localized high pH environment affects bone integration [28,29,31]. In vitro and in vivo models have indicated that the high pH is diffused in the body and that the high pH and high Mg concentration in the environment reduces the fusion of preosteoclasts and inhibits osteoclastogenesis. Moreover, according to previous studies, bone marrow stem cells and periosteal stem cells are differentiated into osteoblast—like cells in environments with a low pH and low Mg concentration and the cells migrate to the implantation site [32,33,34,35].

## 4. Materials and Methods

### 4.1. Study Design

The objective of the present study was to create a construct by using PRF with MC3T3-E1 preosteoblasts, combined with a biodegradable Mg ring, to mimic the cell migration and calcium extracellular matrix generation that occurs during bone repair. Regarding the PRF collected from rabbits, their pore space volume was first increased using the repeated freeze drying method and MC3T3-E1 cells were cultured in the PRF. The large pores of PRF offered MC3T3-E1 cells adequate space for migration and the platelets that were part of the PRF scaffold provided growth factors for osteoinduction. Moreover, the Mg ions released from the Mg ring facilitated osteoinduction. The construct has great potential as a scaffold for bone defect repair due to the engineered osteogenetic environment. The PRF was randomly assigned to the following groups: PRF + Mg, PRF + Ti, LPPRF + Mg and LPPRF + Ti. The minimum sample number was N = 5 per group.

### 4.2. PRF Collection and Analysis

**Figure 12:**
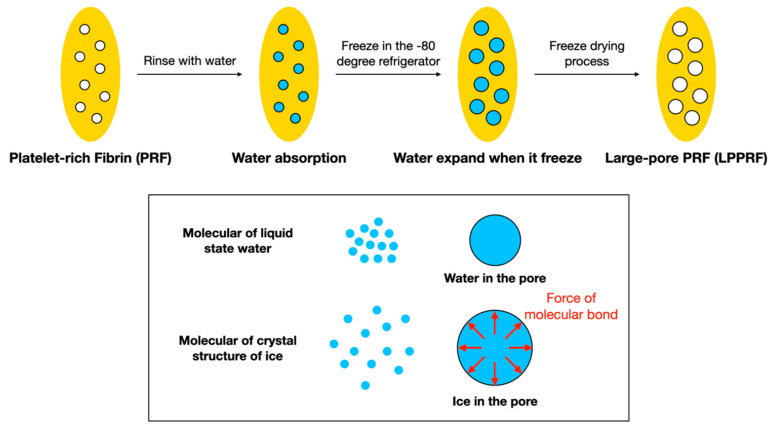
Large-pore platelet-rich fibrin preparation procedure and the molecular basis of why water expands when frozen.

Venous blood was collected from rabbits and stored in a blood collection tube with a clot activator. Centrifugation was then executed in a tabletop centrifuge (Digisystem; Laboratory Instruments, New Taipei, Taiwan). The PRF was self-generated after centrifugation. PRF was subjected to the repeated freeze drying method, to produce a solid scaffold and subsequently subjected to repeated immersion and the repeated freeze drying process was used to enlarge the pore spaces of the PRF scaffold to allow for cell ingrowth; these PRF scaffolds are called LPPRF. To summarize the procedure for engineering LPPRF, the PRF was first frozen in a −80 °C refrigerator overnight to freeze the water, which contained the PRF; freezing the water expanded the pores of PRF due to the differing molecular sizes of liquid water and solid ice (i.e., the density of ice is less than that of water). After the freezing process, the frozen PRF was placed into a freeze dryer (FD4.5-8P-D, Reber’s Instruments, New Taipei, Taiwan) with −60 °C and 0.001 Torr of vacuum for 4 h to remove the moisture. Subsequently, room-temperature DI water was added to the lyophilized PRF for wetting and water absorption by PRF; after 5 min, the extra water was removed using paper tissues and the PRF was placed in a −80 °C refrigerator and freeze dryer again to repeatedly expand the PRF pores (Figure 12). All samples were exposed under ultraviolet light for 24 h for sterilization before use.

### 4.3. Analysis of PRF and LPPRF Structures

The volume, porosity and the volume of pore space of PRF/LPPRF were analyzed through micro-computed tomography (micro-CT) and reconstructed for calculation to ensure that the pore size was suitable for cell ingrowth. After each iteration of the freeze drying process, the PRF or LPPRF were scanned by using micro-CT and were analyzed through the use of CTan analyzer software (Bruker, Billerica, MA, USA); images of the region of interest were selected for calculation. The tissue volume (mm^3^), porosity (%) and volume of pore space (mm^3^) of PRF were calculated according to the chosen region of interest and the intensity of the images were determined by the CTan analyzer software. Each time point of tissue volume, porosity and volume of pore space results were normalized with the data of the first lyophilize cycle.

### 4.4. Growth Factor Tests

Equally weighted PRF and LPPRF samples were immersed in 10 mL of PBS at a temperature of 37 °C for 10 days. During immersion, 100 μL of extraction PBS was collected on days 1, 3 and 10 for growth factor tests by using an ELISA kit. ELISA kits for the selected growth factor, TGF-β, IGF-1 and VEGF, were used for the quantification of the growth factors released from PRF and LPPRF in PBS.

### 4.5. Preparation of Mg and Ti scaffolds

Pure Mg (>99.9% purity) and pure Ti (>99.9% purity) were obtained from a supplier (Gredmann Group, Taipei, Taiwan) and machined into a ring shape with an outer diameter, inner diameter and thickness of 6, 4 and 5 mm, respectively, by using wire electrical discharge machining. The surface of the metallic ring was polished with #2000 sandpaper. The inner diameter was designed to enable the placement of PRF plugs. The samples were then polished on all surfaces to ensure approximately corresponding roughness and profiles across all samples. All samples were exposed under ultraviolet light for 24 h for sterilization before use.

### 4.6. Degradation Behavior of the Metallic Ring

The degradation behavior of pure Mg and Ti manufactured rings was investigated through their immersion in Hank’s solution at a temperature of 37 °C and this method was employed according to the standard ASTM G31-72 [21]. After various immersion periods (0, 1, 2, 4 and 6 weeks), the samples were removed from the Hank’s solution, rinsed with distilled water and then dried at 100 °C in an oven. The weight loss of and pH change in the samples were measured using an electronic balance (Mettler Toledo; AX205 Delta Range, Columbus, OH, USA) and a pH meter (F-52; Kyoto, Japan), respectively.

### 4.7. Surface Morphology Observation of Metallic Ring and MC3T3-E1 Preosteoblasts When Attached to PRF and LPPRF

The metallic ring that was immersed with Hank’s solution and the morphology of MC3T3-E1 preosteoblasts attached on the metallic ring were observed through a scanning electron microscope (SEM; SU3500; Hitachi, Tokyo, Japan). The metallic ring was dried in an oven before being observed using SEM. As for the observation of cell attachment, the PRF and LPPRF were placed in a 24-well culture plate and the cell suspension (300 μL, 2000 cells/well) was dispensed into a 24-well culture plate and incubated for 4 h to ensure that the cells had sufficient time for attachment. After the fixation and dehydration process was complete, the cell morphology was observed and captured using SEM.

### 4.8. Precipitate Medium Preparation

The PRF, LPPRF, Mg ring and Ti ring precipitate media were first prepared for an indirect-contact biocompatibility test, transmembrane cell migration test and calcium deposition test. All samples were immersed in an alpha minimum essential medium (α-MEM) for 7 days and then removed. The media containing the PRF, LPPRF, Mg ring, or Ti ring precipitates were labeled as 100% precipitate media. In further experiments, the precipitate media were diluted to a 100% PRF medium, 100% LPPRF medium, 100% Mg medium, 100% Ti medium, 50% PRF + 50% Mg medium, 50% PRF + 50% Ti medium, 50% LPPRF + 50% Mg medium and 50% LPPRF + 50% Ti medium.

### 4.9. Cell Viability of MC3T3-E1 Preosteoblasts

MC3T3-E1 preosteoblasts were used to analyze the biocompatibility of PRF, LPPRF, Mg rings, Ti rings and their mixtures by performing a 3-(4,5-dimethythiazol-2-yl)-2,5-diphenyl tetrazolium bromide (MTT) assay using an indirect-contact method. MC3T3-E1 osteoblasts were first cultured in alpha-MEM (Gibco, Gaithersburg, MD, USA) supplemented with 10% fetal bovine serum (Gibco). In the MTT assay, 100 μL of cell suspension was first added to a well and incubation was then performed in a 96-well culture plate that had a cell density of 5000 cells/well for 24 h at 37 °C in a 5% CO_2_ atmosphere. After it was preincubated to enable cell attachment, the culture medium was replaced by 100 μL of one of the eight diluted precipitate media for further incubation in the same environment for 3 days. Subsequently, 10 µL of MTT solution (Invitrogen, Carlsbad, CA, USA) was gently added to the well and incubated for 3 h. Subsequently, 100 μL of dimethylsulfoxide was added and the optical density was detected using an ELISA reader at a wavelength of 560 nm (Multiskan FC; Thermo, Waltham, MA, USA).

### 4.10. Analysis of MC3T3-E1 Cell Migration Capacity through a Scratch Assay

The migration capacity of MC3T3-E1 preosteoblasts was analyzed using a scratch assay. The migration capacity of MC3T3-E1 cells was stimulated by eight types of diluted precipitate media: the 100% PRF medium, 100% LPPRF medium, 100% Mg medium, 100% Ti medium, 50% PRF + 50% Mg medium, 50% PRF + 50% Ti medium, 50% LPPRF + 50% Mg medium and 50% LPPRF + 50% Ti medium. The MC3T3-E1 cell suspension (5000 cells) was first added to a six-well culture plate for incubation. After 24 h, a straight line was scratched along the monolayer with the tip of a 1000 μL pipet. Debris was then gently removed with the culture medium. A suitable volume of culture medium was added to the culture plate for incubation. After 4, 8 and 24 h of incubation, the cells were observed and imaged using an optical microscope (Primovert; Zeiss, Jena, Germany). Image J software was used to measure the distance traveled during the desired time frame.

### 4.11. Migration Capacity of MC3T3-E1 Cells in PRF and LPPRF Sheets

To clearly observe the cells’ migration capacity in PRF and LPPRF scaffolds to establish whether the pore size of the LPPRF was adequate, the collected PRF scaffold was cut into 0.3 mg pieces and placed in a stainless-steel die with 3 μm thickness for the execution of the repeated freeze drying process; this process enlarged the pores of the PRF scaffold to create thin PRF and LPPRF sheets. The freeze drying process was executed thrice to generate LPPRF sheets with large pores.

The PRF and LPPRF sheets obtained by performing freeze drying at various numbers of iterations were placed in a channel of two stuck coverslips, with gap heights of 3 μm and gap widths of 2 mm and were created with a slide, cover slip and paraffin. The holder was then placed in a 10 cm culture dish that had undergone well sterilization. PRF and LPPRF sheets were first immersed in the standard culture medium to prewet the membranes. Subsequently, 20 μL of MC3T3-E1 cell suspension was added to the channel and incubation was performed for 2 h. After cell attachment, cells were stained with the DNA-binding fluorescent dye Hoechst 33342 (Sigma-Aldrich; St. Louis, MO, USA). Images of cell migration in the PRF and LPPRF sheets were captured using a fluorescence microscope (Revolve; ECHO, San Diego, CA, USA) in a consistent position at 0, 1, 2, 4, 6 and 8 h after cell seeding.

### 4.12. Extracellular Matrix Calcium Deposition of MC3T3-E1 Cells with Precipitate Medium Culture

MC3T3-E1 cells were used to facilitate extracellular matrix calcium deposition through the diluted precipitate medium, which was prepared by using PRF, LPPRF, Mg rings and Ti rings. First, 1 mL of MC3T3-E1 cell suspension was added to a 48-well culture plate with a cell density of 3000 cells/well and incubated for 24 h by using a standard culture medium. After 24 h, the culture medium was removed and replaced with various types of diluted precipitate medium. After 7 days of incubation, a gentle washing with PBS and the fixing of the cells were performed using 4% paraformaldehyde. Alizarin red S staining dye was then used to stain calcium deposits generated by MC3T3-E1 cells. The samples were observed and images captured using an optical microscope (Primovert; Zeiss, Germany).

### 4.13. Extracellular Matrix Calcium Deposition of MC3T3-E1 Cells on PRF and LPPRF Scaffolds with Mg and Ti Rings

PRF and LPPRF scaffolds produced through repeated freeze drying were used in this experiment. All scaffolds were placed into Mg and Ti rings in a 6 cm dish and prewetted with a culture medium. Subsequently, 5 mL of MC3T3-E1 cell suspension was added to the scaffolds with a cell density of 3000 cells/scaffold and incubated for 7 days. Fixation and dehydration were then executed to complete the sample preparation. Alizarin red S staining was used to stain the calcium deposition on PRF and LPPRF scaffolds. After the completion of 5 μm thick sectioning, the stained images were captured using an optical microscope (Primovert; Zeiss, Germany). The relative quantitative results of stain area were analyzed using Image J software.

### 4.14. Statistical Analysis

All results in the present study are presented as the mean ± standard deviation. All statistical analyses were performed using SPSS 20 (version 20.0, IBM, Armonk, NY, USA). Data were analyzed using the one-way analysis of variance, followed by a post hoc Tukey test and independent-sample *t* test. Statistical significance was set at *p* < 0.05.

## 5. Conclusions

In the present study, we enlarged the pores in the structures of PRFs through repeated freeze drying. LPPRF and a Mg ring were used to create a scaffold for long bone defect applications. Our findings demonstrate that MC3T3-E1 preosteoblasts have a greater migration ability in the PRF structure than in the LPPRF structure. Moreover, the use of a Mg ring enhanced the osteogenic ability and migration capacity of Mg ions during degradation. LPPRF with a Mg ring has much potential in long bone defect repair.

## Data Availability

The data presented in this study are available on request from the corresponding author. The data are not publicly available since they are raw data.
